# Chemical Profiles and Pharmacological Properties with in Silico Studies on *Elatostema papillosum* Wedd

**DOI:** 10.3390/molecules26040809

**Published:** 2021-02-04

**Authors:** Md. Zia Uddin, Arkajyoti Paul, Ahmed Rakib, Saad Ahmed Sami, Shafi Mahmud, Md. Sohel Rana, Shahadat Hossain, Abu Montakim Tareq, Mycal Dutta, Talha Bin Emran, Jesus Simal-Gandara

**Affiliations:** 1 Department of Pharmacy, BGC Trust University Bangladesh, Chittagong 4381, Bangladesh; zia@bgctub.ac.bd (M.Z.U.); arka.bgctub@gmail.com (A.P.); mycal@bgctub.ac.bd (M.D.); 2 Department of Pharmacy, Jahangirnagar University, Savar, Dhaka 1342, Bangladesh; sohelrana.ju@gmail.com; 3 Department of Pharmacy, Faculty of Biological Sciences, University of Chittagong, Chittagong 4331, Bangladesh; rakib.pharmacy.cu@gmail.com (A.R.); s.a.sami18pharm@gmail.com (S.A.S.); 4 Microbiology Laboratory, Bioinformatics Division, Department of Genetic Engineering and Biotechnology, University of Rajshahi, Rajshahi 6205, Bangladesh; shafimahmudfz@gmail.com; 5 Atomic Energy Centre, East Nasirabad, Chittagong 4209, Bangladesh; sahedmc@gmail.com; 6 Department of Pharmacy, International Islamic University Chittagong, Chittagong 4318, Bangladesh; montakim0.abu@gmail.com; 7 Nutrition and Bromatology Group, Department of Analytical and Food Chemistry, Faculty of Food Science and Technology, University of Vigo-Ourense Campus, E32004 Ourense, Spain

**Keywords:** *Elatostema papillosum*, phytochemicals, medicinal plants, traditional medicine, molecular docking

## Abstract

The current study attempted, for the first time, to qualitatively and quantitatively determine the phytochemical components of *Elatostema papillosum* methanol extract and their biological activities. The present study represents an effort to correlate our previously reported biological activities with a computational study, including molecular docking, and ADME/T (absorption, distribution, metabolism, and excretion/toxicity) analyses, to identify the phytochemicals that are potentially responsible for the antioxidant, antidepressant, anxiolytic, analgesic, and anti-inflammatory activities of this plant. In the gas chromatography-mass spectroscopy analysis, a total of 24 compounds were identified, seven of which were documented as being bioactive based on their binding affinities. These seven were subjected to molecular docking studies that were correlated with the pharmacological outcomes. Additionally, the ADME/T properties of these compounds were evaluated to determine their drug-like properties and toxicity levels. The seven selected, isolated compounds displayed favorable binding affinities to potassium channels, human serotonin receptor, cyclooxygenase-1 (COX-1), COX-2, nuclear factor (NF)-κB, and human peroxiredoxin 5 receptor proteins. Phytol acetate, and terpene compounds identified in *E. papillosum* displayed strong predictive binding affinities towards the human serotonin receptor. Furthermore, 3-trifluoroacetoxypentadecane showed a significant binding affinity for the KcsA potassium channel. Eicosanal showed the highest predicted binding affinity towards the human peroxiredoxin 5 receptor. All of these findings support the observed in vivo antidepressant and anxiolytic effects and the in vitro antioxidant effects observed for this extract. The identified compounds from *E. papillosum* showed the lowest binding affinities towards COX-1, COX-2, and NF-κB receptors, which indicated the inconsequential impacts of this extract against the activities of these three proteins. Overall, *E. papillosum* appears to be bioactive and could represent a potential source for the development of alternative medicines; however, further analytical experiments remain necessary.

## 1. Introduction

Depression is the most widespread mental disorder and is recognized to present with heterogenous symptoms associated with a variety of psychological and biological factors [1]. Despite the availability of well-known antidepressant drugs [2], basic neuroscience suggests that the discovery of novel mechanisms may lead to the development of more effective pharmacotherapies, which has resulted in the exploration of new molecules with rapid-onset antidepressant and anxiolytic effects while being associated with fewer side effects than current medications [3]. The International Association for the Study of Pain defines pain as an unpleasant sensory and emotional experience, which is typically associated with actual or potential tissue damage [4]. Inflammation is the multifarious biological reaction of the vascular tissue in response to injurious stimuli, including the presence of pathogens, damaged cells, or irritants. Inflammation can result in the local accumulation of plasmatic exudations and blood cells, resulting in the development of inflammatory diseases, such as various types of arthritis, including rheumatoid and gouty arthritis and shoulder tendinitis [5]. Despite the development of recent therapies, the medical community continues to seek more potent and useful analgesics [6], which has encouraged interest in secondary metabolites derived from plants as potential sources of new drug molecules that are clinically effective. Oxidation, which is initiated by reactive free radical species, is another compelling issue. Free radicals can cause various types of disorders in humans, including central nervous system (CNS) injuries, ischemia, atherosclerosis, reperfusion, cancer, gastritis, AIDS, and arthritis [7]. Recently, plant-derived secondary metabolites have been increasingly analyzed as part of the search for novel free radical scavengers [8]. To reduce the occurrence of free-radical-induced diseases, however, additional information regarding the antioxidant effects of plant-derived substances must be elucidated. Because of these existing limitations, attempts are in progress to explore better replacement of these drugs. In this context, indigenous information can contribute to the development of new drugs from medicinal plants [9,10,11,12,13].

We selected the plant *E. papillosum*, which belongs to the Urticaceae family, for our study. Several Elatostema species are located in the regions of Africa, Asia, Australia, and Oceania. *E. papillosum* is found throughout China, Bhutan, India, and Bangladesh [12]. This species is typically found in the hilly areas of the Chittagong district in Bangladesh [12]. *E. papillosum* was selected because most of the pharmacological activities associated with this plant, including the isolation of bioactive phytochemicals have not been well-investigated. Previous reports state that crushed *E. papillosum* plants are used in traditional medicine for hysteria and abdominal pain treatment. To our knowledge, there have been no reported pharmacological outcomes of *E. papillosum* except the applications for hysteria treatment by local practitioners [12]. In the present study, we aimed to explore the bioactive phytochemicals found in *E. papillosum* using gas chromatography-mass spectrometry (GC-MS). Systemic, guided, separation and identification techniques are essential for revealing the potential bioactive and toxic phytochemicals found in plants, as plants contain a variety of phytochemicals [13]. GC-MS, together with the use of proper detection techniques, represents a vital means for the separation and identification of phytochemicals [14]. GC-MS can be easily used to analyze small, volatile, and thermostable components [15,16,17,18].

Previously, our group investigated the in vivo antidepressant, analgesic, and anti-inflammatory activities and the in vitro antioxidant and antibacterial activities of various fractions of crude methanol *E. papillosum* extracts [19]. The crude methanol extract and its chloroform soluble (CS) fraction showed significant antidepressant, analgesic, and anti-inflammatory activities. The methanol extract and its CS fraction also showed significant DPPH free-radical-scavenging activity, and the methanol extract showed antimicrobial activity. Similarly, the petroleum ether soluble (PES) fraction showed significant antidepressant and anti-inflammatory activities [19].

Because the extracts of this plant showed various beneficial effects, we aimed to correlate these previously reported biological activities, especially the antidepressant, anxiolytic, and antioxidant activities, with the results of a computationally-aided molecular docking study to provide some evidence for the traditional and novel therapeutic applications of *E. papillosum*.

## 2. Results

### 2.1. GC-MS Analysis

**Figure 1:**
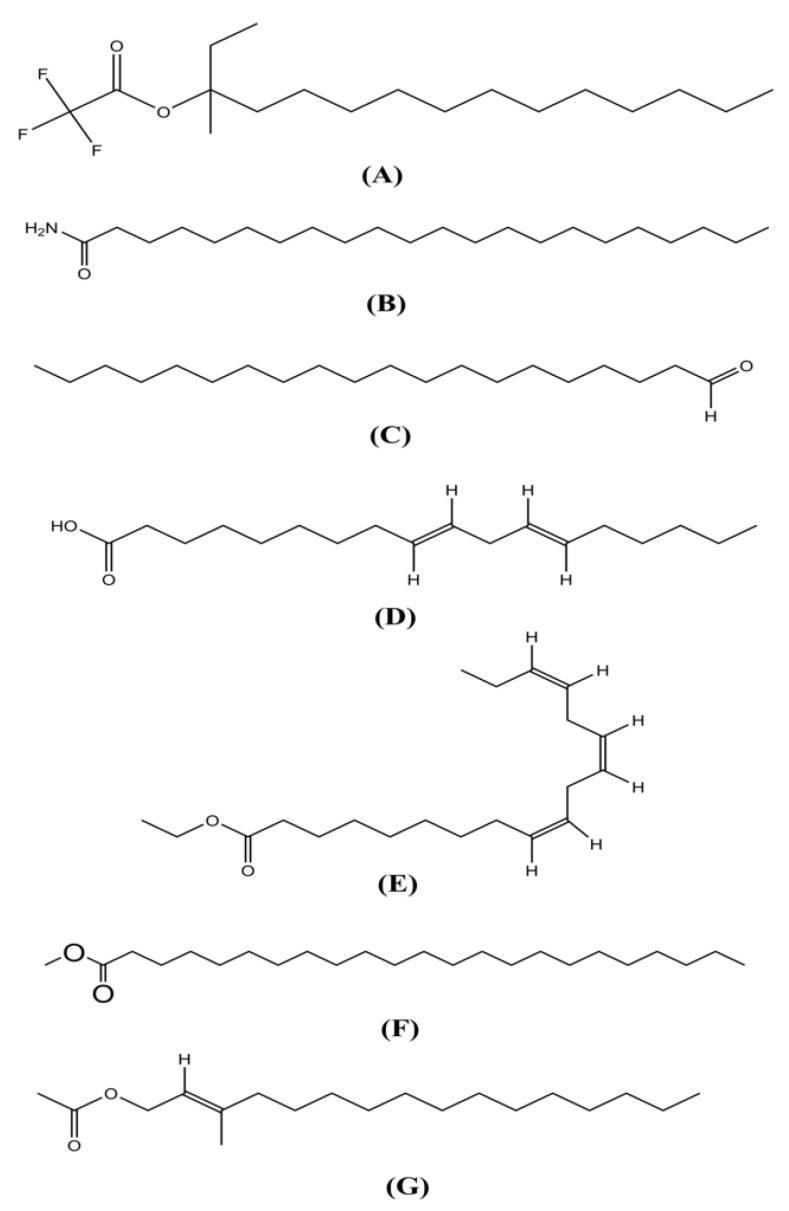
Chemical structures of (**A**) 3-trifluoroacetoxypentadecane, (**B**) 13-docosenamide, (**C**) eicosanal, (**D**) linoelaidic acid, (**E**) linoelaidic acid ethyl ester, (**F**) tricosanoic acid methyl ester, and (**G**) phytol acetate (structures were drawn using ChemDraw Professional version 16.0).

**Figure 2:**
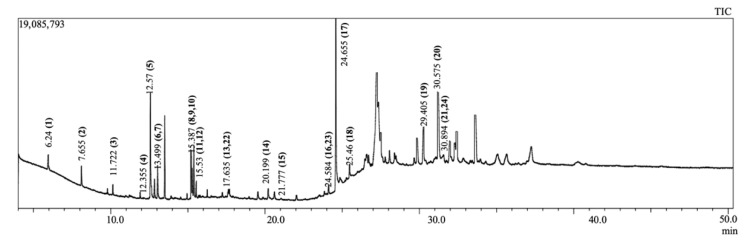
Total ionic chromatogram (TIC) of the whole-plant *E. papillosum* methanolic extract by gas chromatography-mass spectrometry (GC-MS).

A total of 24 compounds were isolated from *E. papillosum* using GC-MS spectroscopy, which are listed in Figure 1 and Table 1, along with their chemical compositions. The total ionic chromatogram (TIC) is shown in Figure 2. Seven compounds were selected for molecular docking analyses because the specific biological activities of interest have not yet been established for these compounds.

### 2.2. Molecular Docking Associated with Antidepressant Activity

**Figure 3:**
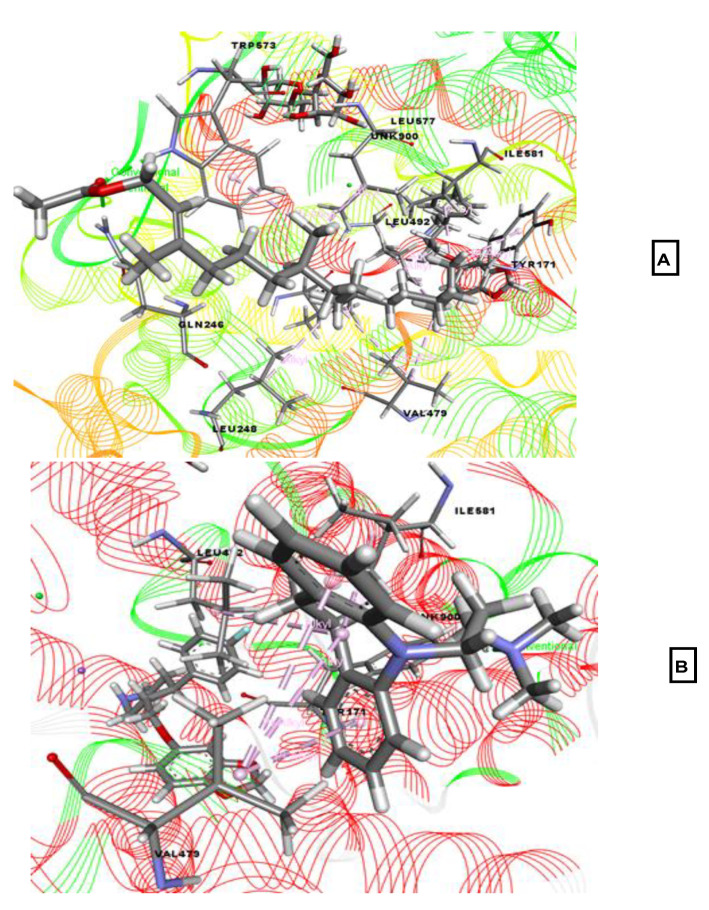
Best-ranked poses of phytol acetate (**A**), and imipramine HCl (**B**), in the binding pocket of the ts3 human serotonin transporter (PDB ID: 5I6X). Green color illustrates the residues forming hydrogen bonds, white color illustrates the residues with carbon–hydrogen interaction and pink color illustrates the residues with hydrophobic (pi-pi/pi-alkyl) stacking.

When examining antidepressant properties, the molecular docking simulation study showed docking score ranges from −3.628 to −0.797 kcal/mol against human serotonin receptor [Protein Data Bank (PDB) ID: 5I6X], and phytol acetate (−3.628 kcal/mol) displayed the highest binding affinity, followed by 3-trifluoroacetoxypentadecane (−3.423 kcal/mol), eicosanal (−2.525 kcal/mol), and linoelaidic acid (−0.797 kcal/mol; Table 2). Imipramine was used as a reference drug for antidepressant activity, which exhibited a docking score of −5.35 kcal/mol, which was greater than all of the selected compounds. In Figure 3, phytol acetate interacted with our target receptor through the formation of hydrogen bonds with Trp573 and Gln246 residues and the formation of hydrophobic bonds with Trp573, Tyr171, Leu577, Ile581, Leu492, Leu492, Val479, Val473, Val488, and Leu248 residues. In contrast, imipramine HCl interacted with Tyr171 through hydrogen bonds and interacted with Val479, Leu492, and Ile581 through hydrophobic bonds.

### 2.3. Molecular Docking Associated with Anxiolytic Activity

**Figure 4:**
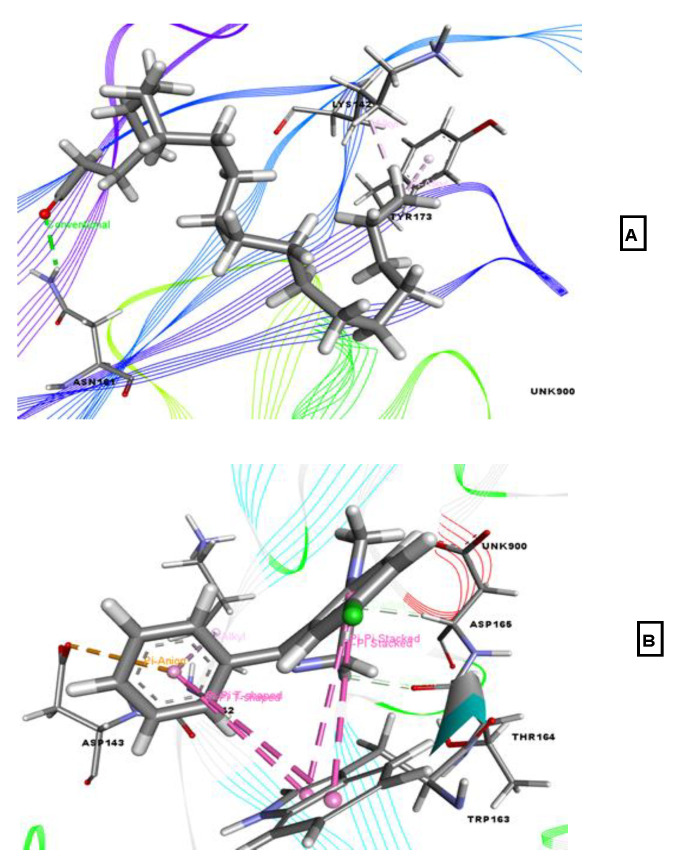
Best-ranked poses for eicosanal (**A**), and diazepam (**B**), in the binding pocket of the KcsA potassium channel (PDB ID: 4UUJ). Green color illustrates the residues forming hydrogen bonds, white color illustrates the residues with carbon–hydrogen interaction and pink color illustrates the residues with hydrophobic (pi-pi/pi-alkyl) stacking.

For the molecular docking simulation study to examine anxiolytic activity, the compounds were docked against the potassium channel receptor (PDB ID: 4UUJ), which resulted in docking scores that ranged from −3.199 to −0.265 kcal/mol. Eicosanal displayed the highest binding affinity (−3.199 kcal/mol), followed by phytol acetate (−2.913 kcal/mol), 3-trifluoroacetoxypentadecane (−2.512 kcal/mol), and linoelaidic acid (−0.265 kcal/mol), as shown in Table 2. The three other phytochemicals did not show any interaction with our target receptor, whereas the reference drug diazepam yielded a docking score of −4.035 kcal/mol. Eicosanal interacted with our target receptor through Asn161, Lys142, and Tyr173 residues. The reference drug diazepam formed various types of hydrophobic bonds with Trp163 (3), Thr164, Asp165, Asp143, and Lys142, as shown in Figure 4.

### 2.4. Molecular Docking Associated with Analgesic Activity

The analgesic potentials of the selected compounds were investigated by performing molecular docking studies using the cyclooxygenase-1 (COX-1; PDB ID: 2OYE) and COX-2 (PDB ID: 6COX) receptors as the target proteins. The docking scores of the selected compounds against the COX-1 receptor ranged from −3.561 to −0.41 kcal/mol. Eicosanal exhibited the best binding affinity (−3.561 kcal/mol) against COX-1, followed by phytol acetate (−3.533 kcal/mol), 3-trifluoroacetoxypentadecane (−3.458 kcal/mol), and linoelaidic acid (−0.41 kcal/mol). The reference drug diclofenac-Na displayed a docking score of −4.59 kcal/mol. Eicosanal interacted with the Leu99 and Leu92 residues of our target protein via hydrophobic bonds. Diclofenac-Na interacted with His90, His95, and Pro514 by forming various hydrophobic bonds and formed two hydrogen bonds with His90 and Pro514.

Additionally, phytol acetate exhibited the best binding affinity (−5.236 kcal/mol) against the COX-2 protein, followed by linoelaidic acid (−2.96 kcal/mol). None of the other compounds interacted with this target protein. The reference drug diclofenac-Na exhibited a docking score of −7.26 kcal/mol. Phytol acetate interacted via hydrophobic bonds with Leu352, Leu359, Leu531, Leu93, Val349, Val89, Pro86, and Tyr115 residues. The reference drug formed hydrogen bonds with Arg120 and various hydrophobic bonds with Val349 (2), Leu352, Ser353, Val523, Gly526, and Ala527 (3).

**Figure 5:**
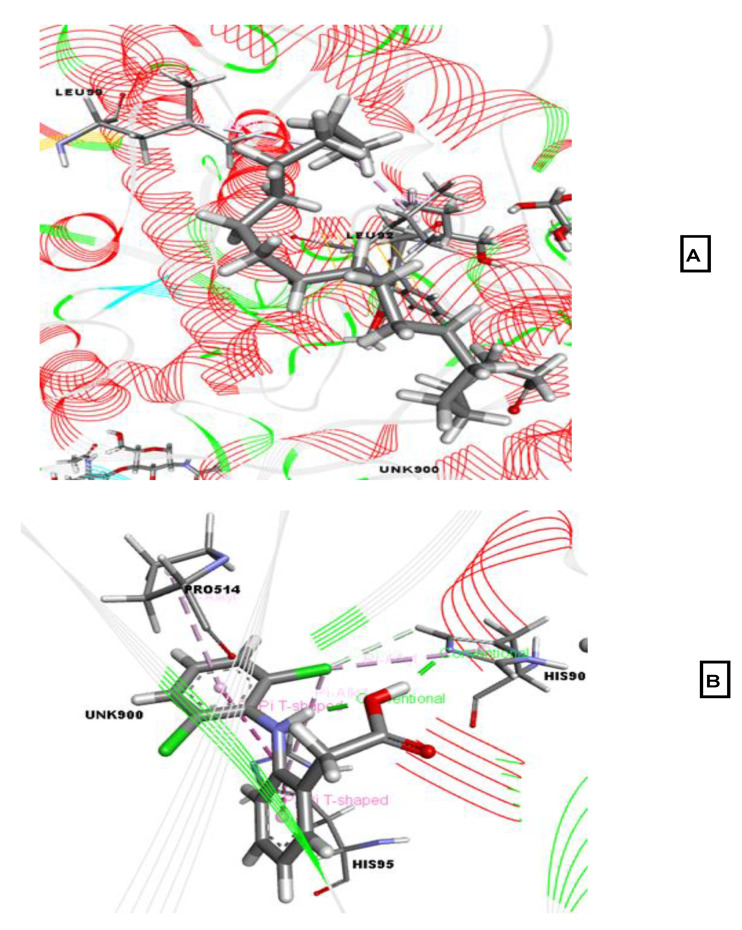
Best-ranked pose of eicosanal (**A**), and diclofenac-Na (**B**), in the binding pocket of COX-1 (PDB ID: 2OYE) enzymes. Green color illustrates the residues forming hydrogen bonds, white color illustrates the residues with carbon–hydrogen interaction and pink color illustrates the residues with hydrophobic (pi-pi/pi-alkyl) stacking.

**Figure 6:**
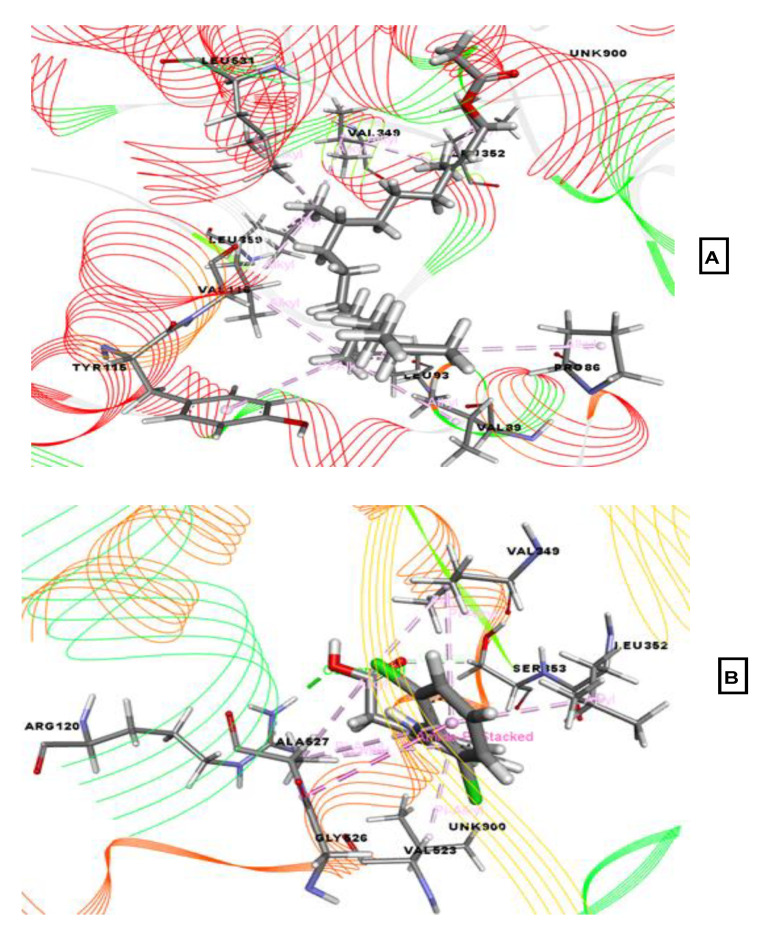
Best-ranked pose of phytol acetate (**A**), and diclofenac-Na (**B**), in the binding pocket of COX-2 (PDB ID: 6COX) enzymes. Green color illustrates the residues forming hydrogen bonds, white color illustrates the residues with carbon–hydrogen interaction and pink color illustrates the residues with hydrophobic (pi-pi/pi-alkyl) stacking.

The results of the docking study are shown in Table 2, and the docking figures are presented in Figure 5 and Figure 6.

### 2.5. Molecular Docking Associated with Anti-Inflammatory Activity

**Figure 7:**
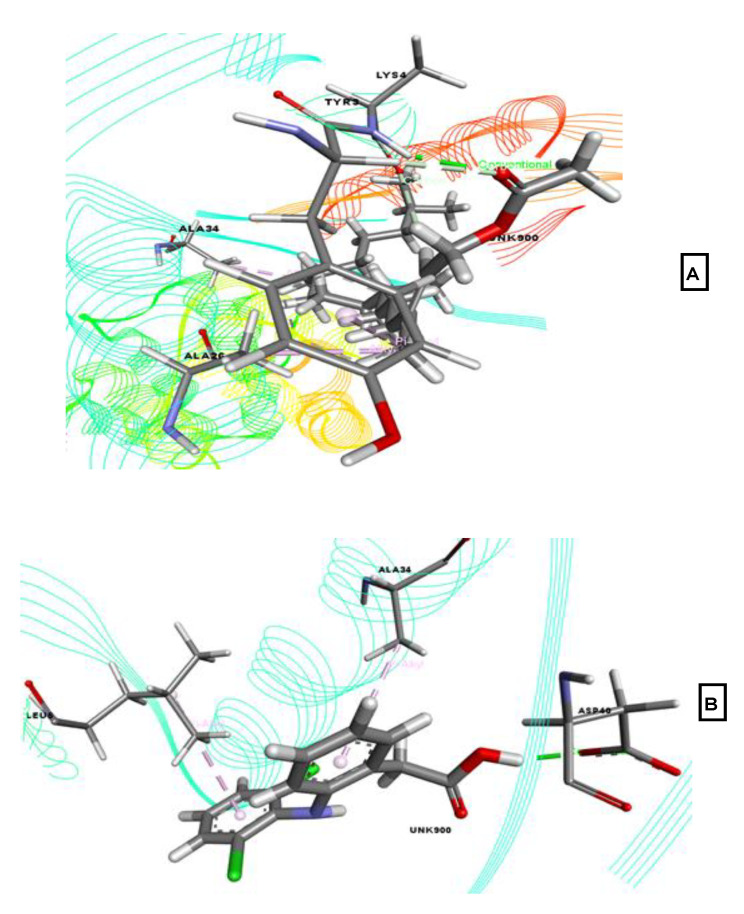
Best-ranked poses for phytol acetate (**A**), and diclofenac-Na (**B**), in the binding pocket of nuclear factor kappa-light-chain-enhancer of activated B-cells (NF-κB; PDB ID: 5LDE). Green color illustrates the residues forming hydrogen bonds, white color illustrates the residues with carbon–hydrogen interaction and pink color illustrates the residues with hydrophobic (pi-pi/pi-alkyl) stacking.

From the data reported in Table 2 it can be observed that, phytol acetate exhibited the highest binding affinity with nuclear factor kappa-light-chain-enhancer of activated B cells (NF-κB; PDB ID: 5LDE), with a score of −4.153 kcal/mol, followed by 3-trifluoroacetoxypentadecane (−4.012 kcal/mol). The reference drug, diclofenac-Na, showed a binding score of −5.758 kcal/mol. However, 13-docosenamide, linoleic acid ethyl ester, and tricosanoic acid methyl ester did not bind with NF-κB. As shown in Figure 7, phytol acetate interacted with the target protein through Ala34, Ala26, Tyr3, and Lys4 residues. Diclofenac-Na formed hydrophobic bonds with Ala34 and Leu5 and a hydrogen bond with Asp40.

### 2.6. Molecular Docking Associated with Antioxidant Activity

**Figure 8:**
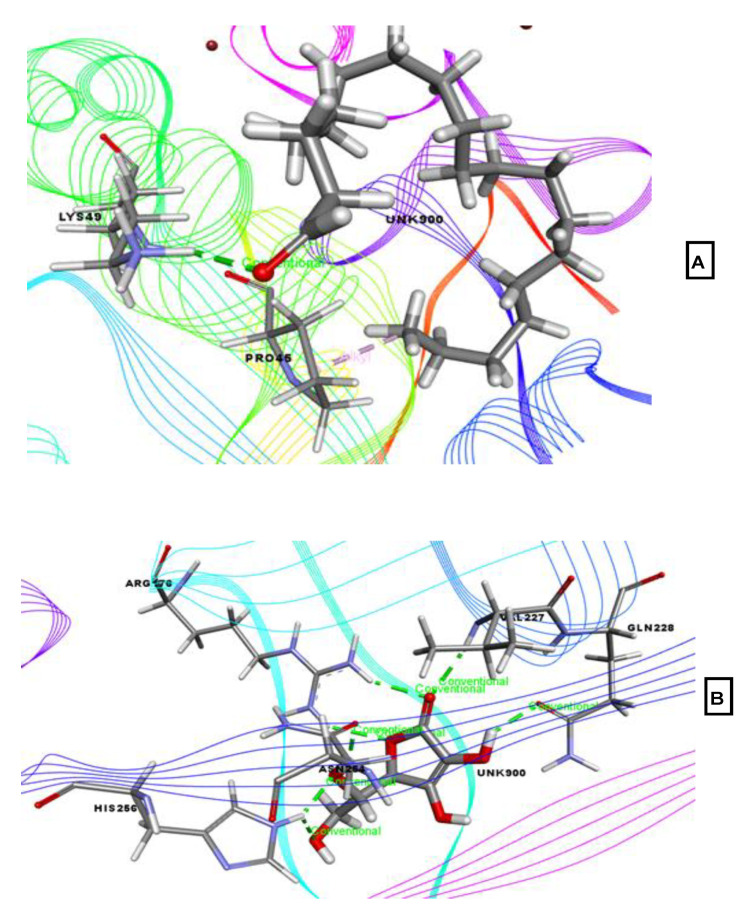
Best-ranked pose for eicosanal (**A**), and ascorbic acid (**B**), in the binding pocket of human peroxiredoxin 5 (PDB ID: 1HD2). Green color illustrates the residues forming hydrogen bonds, white color illustrates the residues with carbon–hydrogen interaction and pink color illustrates the residues with hydrophobic (pi-pi/pi-alkyl) stacking.

Our study revealed a range of docking scores ranging from −3.928 to −1.17 kcal/mol against human peroxiredoxin 5 enzyme (PDB ID: 1HD2) when evaluating potential antioxidant activity (Table 2). Eicosanal possessed the best docking score (−3.928 kcal/mol), followed by phytol acetate (−1.469 kcal/mol), 3-trifluoroacetoxypentadecane (−1.469 kcal/mol), and linoelaidic acid (−1.17 kcal/mol). Eicosanal interacted with the Lys49 residue of the target protein via hydrogen bond and with the Pro45 residue through hydrophobic interaction. However, ascorbic acid, which was used as a reference drug for antioxidant activity, exhibited the highest docking score of −5.134 kcal/mol, interacting with Arg176 (2), His256 (2), Asn254, Val227, and Gln228 via hydrogen bonds (Figure 8).

### 2.7. Ligand-Based ADME/T Predictions

The seven selected bioactive compounds were screened for drug-candidacy by evaluating their pharmacokinetic and physicochemical properties using the QikProp module of Schrödinger Suite-Maestro, version 10.1. The range of different ADME (absorption, distribution, metabolism, and excretion) parameters that were evaluated included the molecular weight, the estimated number of hydrogen bond donors (HB donors) and acceptors (HB acceptors) in the solute when combined with water molecules in an aqueous solution, and the total solvent accessible surface area (SASA) value. These values for all compounds were found to be within the acceptable ranges. These compounds also demonstrated good brain/blood partition coefficient (QPlogBB) values, ranging from −1.523 to 0.25, which suggested that the compounds are within acceptable limits. The values of the octanol/water partition coefficient (QPlog Po/w) ranged from 4.826 to 7.528. The aqueous solubility (QPlogS) values for the selected compounds ranged from −13.069 to −5.802. All of the selected compounds fulfilled Lipinski’s rule of five (RO5), with none of the compounds contravening more than one of Lipinski’s rules (Table 3). The selected compounds displayed high percentages of human oral absorption, with all exceeding 80%, and most of the phytochemicals exhibited 100% oral absorption.

### 2.8. Molecular Mechanics Generalized Born Surface Area (MM-GBSA) Analysis and Ligand Efficiency

In Table 4, the compounds showing MM-GGBSA ΔG bind against particular receptors. There was a good correlation between MMGBSA ΔG bind and binding affinity. We also report on the use of an in silico approach to predict the binding affinities and ligand efficiencies of *E. papillosum* constituents towards distinct receptors.

### 2.9. Molecular Dynamics Simulations

**Figure 9:**
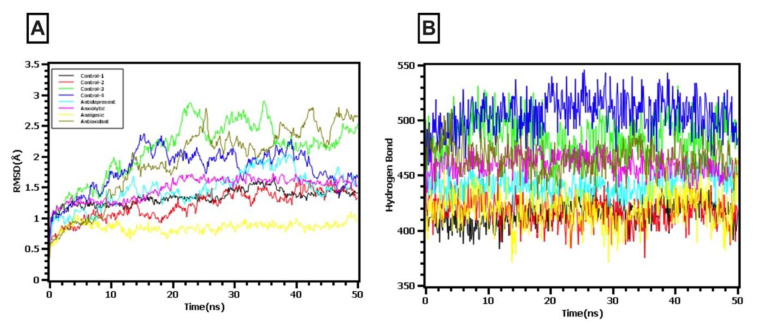
The molecular dynamics simulation study of the docked complex. (**A**) Root mean square deviation (RMSD), and (**B**) hydrogen bond patterns were assessed from the simulation trajectories. Phytol acetate (5I6X) was compared against imipramine HCl (control 1), eicosanal (4UUJ) was compared against diazepam (control 2), phytol acetate (5LDE) was compared against diclofenac-Na (control 3), and eicosanal (1HD2) was compared against ascorbic acid (control 4).

The root-mean-square deviations of the drug–protein complexes were explored to examine the steady-state characteristics of the complexes. Figure 9A demonstrates that the compound with the best analgesic activity did not display any fluctuations throughout the entire simulation time, exhibiting a steady conformation. The control 1 antioxidant and anxiolytic and control 2 showed less deviation, although they initially displayed a high level of flexibility subsequently but stabilized. The control 3 and analgesic complexes displayed larger changes during the initial phase but did not exceed an RMSD value above 2.5 Å. The RMSD descriptors from eight complexes in Figure 9A correlates with the stability of the complexes.

The hydrogen bond formations observed during the simulation define the overall integrity of the complexes. A higher degree of changes observed in the hydrogen bond patterning indicates complex instability and increased flexibility. The hydrogen bonds depicted in Figure 9B showed that all eight complexes between the tested compounds and the target protein displayed stable hydrogen bonding patterns, with no significant changes in the hydrogen bond numbers observed, suggesting that all eight complexes maintained structural integrity.

## 3. Discussion

Recently, plant-derived substances have garnered significant interest because of their remarkable applications. Medicinal plants are the richest bioresources for conventional drugs, modern medicines, nutraceuticals, curative intermediates, and artificial drug chemicals. Medicinal plants have also been used by human society to combat diseases since the dawn of civilization. A large portion of the population, especially those who live in rural communities, largely depends on herbal remedies. Several herbal remedies have withstood the test of time, particularly those used to treat allergic, metabolic, and degenerative diseases associated with aging. However, scarce scientific data regarding the chemical identities and effectiveness of these herbs are available, except those associated with the practice of Ayurveda and Unani medicine [8,13]. In this study, we conducted a correlation study between our previously reported bioactivities and a computer-aided molecular docking study to provide evidence to support the traditional and novel therapeutic use of *E. papillosum*. Although phytochemicals obtained from medicinal plants were focused on the empiric experience in the past, currently, the scientific evidence regarding the chemical composition and therapeutic properties are regarded as the main focus while isolating several phytochemicals. Expert taxonomists mainly perform the identification and authentication of medicinal plants; however, one of the main disadvantages includes the absence of several phenotypic characteristics. In addition, the products used in traditional medicine are processed in various forms, such as powder, extracts, capsules, and tablets. Phytochemical characterization could therefore be used in the identification and authentication of several medicinal plants [20,21,22,23,24,25].

Molecular docking has become a widely employed tool in the field of computer-aided drug design because molecular docking allows for binding predictions to be made between small compounds and large macromolecules and various target proteins [26]. Molecular docking can also be utilized to understand the probable mechanisms of action for different pharmacological activities [27]. Several studies have recently used molecular docking analysis to predict the binding mode of numerous phytochemicals obtained from several medicinal plant extracts with the respective receptors for various pharmacological activities [17,28]. In our present study, we implemented a molecular docking analysis to explore the binding affinities of selected compounds against various target proteins. Eicosanal and phytol acetate are the two compounds that have displayed the best interactions with a variety of target proteins to exert a various pharmacological activities (antioxidant, antidepressant, anxiolytic, analgesic, and anti-inflammatory activities). Eicosanal displayed the highest binding affinity against the target proteins examined to assess analgesic, anxiolytic, and antioxidant activity, whereas phytol acetate displayed the best docking scores against the target proteins examined to assess analgesic, anti-inflammatory, and antidepressant activity.

Pharmacologically, COX-1 and COX-2 are responsible for the formation of prostanoids, which act in several pathways [29,30]. In general, the synthesis of prostanoids is mediated by phospholipase A_2_, which regulates the release of arachidonic acid from membrane phospholipids [4,31]. Therefore, COX inhibition correlates not only with the pharmacological inhibition of various downstream biochemical effects but also inhibits the synthesis of prostaglandins (PGs) [31]. In our current study, the targeted compounds interacted with both COX-1 and COX-2; however, the interactions were weaker than that for the positive control used in the study. Despite having a lower docking score, eicosanal interacted with the Leu92 residue through hydrophobic interaction. A recent study documented that the Leu92 residue is responsible for stabilizing the structure. Similar results were observed during the interaction with COX-2 and the targeted compounds, for which diclofenac-Na possessed the highest docking score. However, phytol acetate interacted with COX-2 by forming a hydrophobic interaction with several residues, including Leu352, Leu359, Leu531, Leu93, Val349, Val89, Pro86, and Tyr115. A previous study by Krisnamurti et al. reported that acetaminophen binds with Leu352 and Val349 through hydrophobic interactions and may interfere with the COX-2 activity [32]. In addition, NF-κB has been viewed as a prominent target for anti-inflammatory and analgesic responses [33,34]. In the present experiment, phytol acetate interacted with NF-κB with the greatest affinity. Previously, NF-κB inhibition was shown to be mediated by phytol acetate found in the hexane extract of *Cymbopogon citratus* [35].

To perform the in silico analysis of potential anxiolytic and antidepressant activities, we selected the potassium channel receptor and the human serotonin receptor, respectively. Potassium channels regulate the excitability of neurons, and the serotonin receptor is strongly associated with the etiology and pharmacology of depression [36,37]. Our previous study demonstrated the inhibition of both receptors by selective phytocompounds isolated from *Piper sylvaticum* plant extract [38]. Moreover, we also analyzed the antioxidant effects of the selected phytocompounds by performing a molecular docking analysis using the human peroxiredoxin 5 enzyme. Here, eicosanal interacted with the Lys49 residue by forming a hydrogen bond, which agrees with the results of a previous study by Bharati et al. [39].

In addition to molecular docking simulations, ADME/T analyses were performed. In the recent era, drug design and discovery approaches have shifted from phenotypic screens to high throughput screening and combinatorial chemistry. Thus, the physicochemical properties are concerned as remarkable characteristics during the selection of drug molecules, which displayed a shift towards higher molecular weight and lipophilicity. The ADME/T analysis using the “rule of five” (RO5) is currently recognized as a widespread marker during drug designing that depicts the solubility profile and permeability. The selected phytocompounds were further analyzed using the QikProp module of the Schrödinger suite-Maestro, version 10.1, to explore their drug-likeness behaviors and their physicochemical and pharmacokinetic characteristics. Compounds that contravene any of Lipinski’s RO5 may encounter problems due to permeability, absorption, and bioavailability, as ligand molecules with lower molecular weights, hydrogen bond capacities, and lipophilicity typically exhibit better permeability [40], faster absorption, and higher bioavailability [41,42]. According to Lipinski’s RO5, our selected phytocompounds displayed good orally-active drug-candidacy profiles. Additionally, assessments of the abilities of the drugs to pass through the blood–brain barrier (QPlogBB), the total SASA, and the percent human oral absorption also indicated that these compounds might be considered as potential drug molecules with receptor-based optimization.

## 4. Materials and Methods

### 4.1. Preparation of Crude Methanolic Extract

Whole plant parts of *E. papillosum* were collected from Chittagong district (22°36′00″ N, 91°40′14.13″ E) of Bangladesh. The plants were sun dried for several days and then oven dried for 24 h at considerably low temperature (below 40 °C) to facilitate grinding. The powdered material (500 g) was macerated in 2.5 L of methanol for 15 days and then filtered through a cotton plug followed by Whatman filter paper number 1. The extract was concentrated with a rotary evaporator (RE 200, Bibby Sterling Ltd., UK) at low temperature (40–45 °C) and reduced pressure. The viscous mass was stored in a refrigerator (4 °C) for future use.

### 4.2. Gas Chromatography-Mass Spectrometry (GC-MS) Analysis

The methanolic leaf extract of *E. papillosum* was inspected using a mass spectrometer (TQ 8040, Shimadzu Corporation, Kyoto, Japan) using the electron impact ionization method, and a gas chromatograph (GC-17A, Shimadzu Corporation) fused with silica capillary column (Rxi-5 ms; 0.25 m film, 30 m long, and internal diameter: 0.32 mm) coated with DB-1 (J&W). The oven temperature was programmed as 70 °C (0 min) increasing to 150 °C, at 10 °C/s. with a hold time of 10 min. A 260 °C temperature was maintained as the inlet temperature. The flow rate was set to a speed of 0.6 mL/min, using helium gas at a 90 kPa constant pressure. The interface temperature from GC to MS was maintained at a constant 280 °C. The MS was set in scan mode with a scanning range of 40–350 amu; the ionization mode was EI (electron ionization) type. The injection volume of the sample was one microliter. The entire GC-MS procedure lasted for 50 min [31]. A comparison with the National Institute of Standards and Technology (NIST) GC-MS library version 08-S was performed to identify the compounds in the peak areas

### 4.3. Computational Molecular Docking Analysis

#### 4.3.1. Chemical Compounds Studied

Seven bioactive compounds were selected for molecular docking analysis, and the PubChem database (https://pubchem.ncbi.nlm.nih.gov (accessed on 7 February 2021)) was used to download the structures of the compounds. The selected compounds were 3-trifluoroacetoxypentadecane (PubChem CID: 534406), 13-docosenamide, (Z)-(PubChem CID: 5365371), linoelaidic acid (PubChem CID: 5282457), linoleic acid ethyl ester (PubChem CID: 5282184), eicosanal (PubChem CID: 75458), phytol acetate (PubChem CID: 6428538), and tricosanoic acid methyl ester (PubChem CID: 75519) (see Figure 1).

#### 4.3.2. Preparation of Ligand

The chemical structures of the seven compounds that were isolated by GC-MS analysis were downloaded from PubChem. The structures were neutralized at pH 7.0 ± 2.0 and minimized by force field OPLS 2005 embedded in Schrödinger Suite-Maestro, version 10.1.

#### 4.3.3. Preparation of Receptor/Enzymes

Three-dimensional crystallographic enzyme structures were obtained from the PDB [43]: KcsA potassium channel (PDB ID: 4UUJ), ts3 human serotonin transporter (PDB ID: 5I6X) [44], human peroxiredoxin 5 (PDB ID: 1HD2) [45], NF-κB (PDB ID: 5LDE), COX-1 (PDB ID: 2OYE) and COX-2 (PDB ID: 6COX) [46,47,48,49]. The enzymes were prepared for the docking experiment by the Protein Preparation Wizard embedded in Schrödinger Suite-Maestro, version 10.1.

#### 4.3.4. Glide Docking

A molecular docking study was performed to interpret the potential mechanisms of the selected compounds against several suitable proteins associated with anxiolytic, antidepressant, antioxidant, analgesic, and anti-inflammatory activities. Docking analysis was performed using Maestro by standard precision scoring function, as previously described [50,51].

### 4.4. Ligand-Based Pharmacokinetic Parameter Analysis

The QikProp module of the Schrödinger Suite-Maestro, version 10.1, is a quick, precise, easy-to-use, online-based program designed to predict significant pharmacokinetic and physicochemical descriptors linked to ADME properties. QikProp evaluates the admissibility of ADME properties to ascertain the drug-likeness of selected ligand molecules, based on Lipinski’s RO5. The ADME/T properties of the selected bioactive compounds (3-trifluoroacetoxy pentadecane, 13-docosenamide, linoelaidic acid, linoleic acid ethyl ester, eicosanal, phytol acetate, and tricosanoic acid methyl ester) were analyzed using the QikProp 3.2 module [52].

### 4.5. Prime Molecular Mechanics Generalized Born Surface Area (MM-GBSA) and Ligand Efficiency

Prime MM-GBSA approach was used to calculate ligand-binding energies and ligand strain energies for a ligand and a receptor. The Prime MM-GBSA module from the Schrodinger software package was utilized to calculate the binding affinity. MM-GBSA is a method that amalgams OPLS 2005 molecular mechanics energies (EMM), an SGB solvation model for polar solvation (GSGB), and a nonpolar solvation term (GNP) composed of the nonpolar solvent surface area and Van der Waals interactions. The higher degree of rigidity of the ligand attached protein is indicated by the higher negative MM-GBSA value. The Prime MM-GBSA process consists of three different approaches; OPLS molecular mechanics energies, an SGB solvation model, and a nonpolar solvent [53]. The binding free energies were calculated from the following equations:

ΔG_bind_ = G_complex_ − (G_protein_ + G_ligand_), (1)

where

G = EMM + VSGB + GNP.(2)

Therefore, to perceive their rigidity along with motion and structural stability in simulation conditions, the best three ligands are selected for further processing. For each ligand, the ligand efficiency was also calculated using the ratio of ΔG to the number of heavy atoms (NHA) for each ligand, ligand efficiency = −(ΔG)/NHA).

### 4.6. Computational Molecular Dynamic Simulations Analysis

The molecular dynamics simulation of the docked complexes was analyzed in YASARA dynamics to evaluate the conformational variations [54]. The AMBER14 force field [55] was used, and the complexes were initially cleaned and optimized. The Particle Mesh Ewald method was applied to calculate the long-range electrostatic interactions [56]. The periodic boundary condition was maintained, and the system was neutralized with the addition of 0.9% NaCl, 7.4 pH at a temperature of 36 °C. The Berendsen thermostat was used to maintain the temperature of the simulation cell [57]. The simulation cell was extended 20Å beyond the complexes, providing more flexibility. The normal simulation time step of 1.25 fs was maintained. The simulation trajectories were saved after every 100-ps interval, and the simulation was run for 50-ns to analyze RMSD and root mean square fluctuation (RMSF) [58,59]. Imipramine HCl, diazepam, diclofenac-Na, and ascorbic acid were denoted as control 1, control 2, control 3, and control 4, respectively.

## 5. Conclusions

To the best of our knowledge, this is the first report describing an in silico correlation between the predicted pharmacological activities of *E. papillosum* and the chemical compounds that characterize its methanolic extract. However, further studies remain necessary to elucidate the mechanisms underlying these effects. Previously, no data regarding this plant has been published; therefore, we believe that *E. papillosum* may be an exemplary sample for alternative therapeutic sources. It is necessary to characterize the structures of secondary metabolites found in the plant and to highlight novel compounds as potential therapeutic components. Thus, this contemporary research can offer some preliminary pharmacological evidence for the ethnomedical uses of *E. papillosum* and it reveals that this plant contains some active agents that may be responsible for these activities.

## Figures and Tables

**Table 1:** Quantitative compounds identified from the *E. papillosum* methanol extract using gas chromatography-mass spectrometry (GC-MS) analysis.

Sl No.	Name	RT	m/z	Area	PA (%)	Molecular Formula	Nature
1	3-butynoic acid	6.24	40	53,887	0.282064878	C_4_H_4_O_2_	Monocarboxylic acid
2	Allene	7.655	40	116,762	0.611176337	C_3_H_4_	Dienes
3	Sebacic acid	11.722	40	36,746	0.19234242	C_16_H_34_O_4_Si_2_	Fatty acids
4	9-octadecen-1-ol	12.355	40	73,511	0.384784294	C_18_H_36_O	Fatty alcohol
5	Phytol acetate	12.57	81	705,294	3.691774752	C_22_H_42_O_2_	Diterpene
6	Hexadecanoic acid, methyl ester	13.499	74	3,180,878	16.64991492	C_17_H_34_O_2_	Fatty acid
7	Pentadecanoic acid, 14-methyl-, methyl ester	13.499	74	3,180,878	16.64991492	C_17_H_34_O_2_	Fatty acid
8	Linoleic acid ethyl ester	15.387	44	90,498	0.473700657	C₂₀H₃_6_O₂	Fatty acid
9	Linoelaidic acid	15.387	44	90,498	0.473700657	C₁₈H₃₂O₂	Fatty acid
10	Phytol	15.387	44	90,498	0.473700657	C_20_H_40_O	Diterpene
11	Heptacosanoic acid, methyl ester	15.53	74	546,048	2.858221139	C_28_H_56_O_2_	Fatty acid
12	Tricosanoic acid methyl ester	15.53	74	546,048	2.858221139	C_24_H_48_O_2_	Fatty acid
13	3-trifluoroacetoxypentadecane	17.635	44	63,831	0.334115524	C_17_H_31_F_3_O_2_	Ester
14	Hexadecanoic acid, 2-hydroxy-1-(hydroxymethyl)ethyl ester	20.199	44	81,277	0.425434466	C_19_H_38_O_4_	Fatty acid glycerol ester
15	Epinephrine	21.777	44	40,058	0.209678677	C₉H₁₃NO₃	Alkaloid
16	Cis-11-eicosenamide	24.584	59	4,682,201	24.50840562	C_20_H_39_NO	Amide
17	2-(16-acetoxy-11-hydroxy-4,8,10,14-tetramethyl-3-oxohexadecahydrocyclopenta[a]phenanthren-17-ylidene)-6-methyl-hept-5-enoic acid, methyl ester	24.655	207	160,560	0.840431585	C_32_H_48_O_6_	Fatty acid
18	3-ethoxy-1,1,1,5,5,5-hexamethyl-3-(trimethylsiloxy)trisiloxane	25.46	207	231,720	1.212909858	C_17_H_50_O_7_Si_7_	Silicate
19	1,2-bis(trimethylsilyl)benzene	29.405	207	37,161	0.194514687	C_12_H_22_Si_2_	Organic compound
20	Stigmasterol	30.575	207	93,362	0.488691913	C₂₉H₄₈O	Phytosterols
21	Beta-sitosterol	30.894	207	126,310	0.66115417	C_29_H_50_O	Phytosterols
22	Eicosanal	17.635	44	67,933	0.355586939	C_20_H_40_O	Aldehyde
23	13-docosenamide	24.584	59	4,682,201	24.50840562	C_22_H_43_NO	Amide
24	Gamma-sitosterol	30.894	207	126,310	0.66115417	C_29_H_50_ O	Phytosteroids

RT: retention time; m/z: m stands for mass and z stands for the charge number of ions; PA: peak area; MW: molecular weight; FAME: fatty acid methyl ester.

**Table 2:** Docking score of the selected compounds identified from the *E. papillosum* methanol extract against the ts3 human serotonin transporter (PDB ID: 5I6X), potassium channel receptor (PDB ID: 4UUJ), COX-1 (PDB ID: 2OYE), COX-2 (PDB ID: 6COX), NF-κB (PDB ID: 5LDE), and human peroxiredoxin 5 receptor (PDB ID: 1HD2) for antidepressant, anxiolytic, anti-inflammatory, analgesic, and antioxidant activity, respectively.

Compounds Name	Docking Score (kcal/mol)					
	5I6X	4UUJ	COX-1	COX-2	5LDE	1HD2
3-trifluoroacetoxypentadecane	−3.423	−2.512	−3.458	-	−4.012	−1.469
13-docosenamide	-	-	-	-	-	-
Linoelaidic acid	−0.797	−0.265	−0.410	−2.960	−1.194	1.170
Linoleic acid ethyl ester	-	-	-	-	-	-
Eicosanal	−2.525	**−3.199**	**−3.561**	-	2.696	**−3.928**
Phytol acetate	**−3.628**	**−2.913**	−3.533	**−5.236**	**−4.153**	−1.469
Tricosanoic acid methyl ester	-	-	-	-	-	-
Standard (Imipramine HCl/ Diazepam/ Diclofenac-Na/ Ascorbic acid)	**−5.350**	**−4.035**	−4.590	−7.260	**−5.758**	**−5.134**

Bold text indicates the best docking score.

**Table 3:** ADME and drug-likeness properties of 3-trifluoroacetoxypentadecane, 13-docosenamide, linoelaidic acid, linoelaidic acid ethyl ester, eicosanal, phytol acetate, and tricosanoic acid methyl ester, as determined by QikProp.

Compound Name	MW ^a^	HB Donors ^b^	HB Acceptors ^c^	SASA^d^	QPlogPo/w ^e^	QPlogBB ^f^	QPlogS ^g^	%Human Oral Absorption ^h^
3-trifluoroacetoxy pentadecane	324.426	0	2	721.905	6.548	−0.338	−7.253	100
13-docosenamide	337.588	2	3	864.813	5.197	−0.461	−9.339	88.609
Linoelaidic acid	280.45	1	2	717.95	5.83	−1.523	−6.373	90.462
Linoleic acid ethyl ester	308.503	0	3	670.733	4.826	0.25	−7.357	100
Eicosanal	296.535	0	2	838.966	6.518	−0.086	−11.068	100
Phytol acetate	338.573	0	2	681.355	6.661	−0.623	−5.802	100
Tricosanoic acid methyl ester	368.642	1	3	977.143	7.528	0.138	−13.069	100

^a^ Molecular weight (acceptable range: <500). ^b^ Hydrogen bond donor (acceptable range: ≤5). ^c^ Hydrogen bond acceptor (acceptable range: ≤10). ^d^ Total solvent accessible surface area using a probe with a 1.4 radius (acceptable range: 300–1000). ^e^ Predicted octanol/water partition coefficient (acceptable range: −2–6.5). ^f^ Predicted blood–brain partition coefficient (acceptable range: −3–1.2). ^g^ Predicted aqueous solubility, S in mol/dm^−3^ (acceptable range: −6.5–0.5). ^h^ Predicted human oral absorption on 0 to 100% scale (<25% is poor and >80% is high).

**Table 4:** Binding affinity (kcal/mol) and ligand efficiencies estimation of the best docked compounds and the standard drugs.

Compound Name/Standard	MM-GBSA ΔG Bind	Ligand Efficiency
**Antidepressant Activity**		
Phytol acetate	−72.00	3.00
Imipramine HCl (Standard)	−49.18	2.24
**Anxiolytic Activity**		
Eicosanal	−42.06	2.00
Phenobarbital (Standard)	−30.65	1.80
**Anti-inflammatory Activity Cyclooxygenase-1 (COX-1)**		
Eicosanal	−47.86	2.28
Diclofenac-Na (Standard)	−37.32	1.87
**Cyclooxygenase-2 (COX-2)**		
Phytol acetate	−75.25	3.14
Diclofenac-Na (Standard)	−54.64	2.73
**NF-κB**		
Phytol acetate	−88.59	3.69
Diclofenac-Na (Standard)	−52.91	2.65
**Antioxidant Activity**		
Eicosanal	−48.99	2.33
Ascorbic acid (Standard)	−35.54	2.96

## Data Availability

Available data are presented in the manuscript.
